# News and Notes

**Published:** 1903-10

**Authors:** 


### *NEWS AND NOTES.*

The attention of doctors and medical students interested in medical college instruction the ensuing fall and spring is called to the card of the University of Louisville.

---

At a baby show in Paris last week the judges decided that out of 150 none was healthy enough to take a prize. The mothers assaulted the judges, and all hands, including the babies, landed in the police station.

---

The Naval Hospital, at Portsmouth, Va., is to be enlarged, its two hundred beds being increased to five hundred. Two modern wings will be built in the rear of the present structure and these will be connected by a third.

---

Dr. Lorenz's first patient in New Orleans, La., a child, we learn from Dr. C. A. Gaudet, of 711 Howard avenue, of that city, has made a perfect recovery. The case was one of talipes equino-varus, and the apparatus was in place for four months.

---

Dr. William A. White, first assistant physician of the Binghamton State Hospital, has been appointed superintendent of the Government Insane Hospital at Washington in place of the late Dr. Alonzo B. Richardson. Dr. White is but thirty-three years old.

---

A Hospital, to cost $75,000, is to be erected in Chicago, the physicians of which must use no alcohol in their prescriptions. The hospital will be called the Frances E. Willard National Temperance Hospital. The three schools of practice—regular, homeopathic, and eclectic—will be represented on the medical staff.

DR. SAMUEL E. EARP has severed his connection with *The Medical and Surgical Monitor*, of Indianapolis, of which he has been editor since 1898. Its career has been successful, and for several years the *Monitor* has had a large subscription list, and is founded upon a sound financial basis. A new editorial force will probably be selected from members of the faculty of the Central College of Physicians and Surgeons.

---

RADIUM is not an impressive substance to the layman. There is a small quantity of it on exhibition at the New York Museum of Natural History, and it looks like a small pinch of light gray snuff in a glass vial. It is a marvelous power, of course, but as it lies in the carefully guarded case it does not look as if it had even a sneeze in it. It is the most disappointing thing that ever had itself announced to draw a crowd.

---

GOVERNOR LANHAM, of Texas, has instructed the State health department to use every means at its command to prevent a spread of the yellow fever in Texas. The people of Laredo are panic stricken over the appearance of the disease at Nuevo Laredo, situated on the opposite bank of the Rio Grande, and many of them are fleeing to other towns. The force of quarantine inspectors at all Texas border points has been increased.

---

THE directors of the Memphis Medical College have established a lectureship, named in memory of the late William E. Rogers, M.D., founder of that institution. The lectureship was established upon the suggestion of the faculty. Once during each session a lecture will be given by a specialist in some branch of medical science, at the invitation of the faculty. The first lecture will be given about the middle of November, 1903, by Dr. Charles H. Hughes, of St. Louis. Dr. Hughes is a professor in the medical department of Washington University.

THE President of the American Congress on Tuberculosis, to be held in Washington, D. C., April 4, 5 and 6, 1905, announces Alfred Meyer, of New York City, consulting physician to the Bedford Sanitarium for Consumptives, to be chairman of a committee in charge of the section on sanitarium treatment of tuberculosis. It is probable that the climatic and other methods of treatment will be comprised under the work of this committee.

---

The following physicians, chosen from last year's graduating class, assumed positions on the house staff of Johns Hopkins Hospital, on the first instant: Frank Goldborough, F. H. Watson, C. W. Young, A. D. Hirschfelder, A. R. Stevens, W. B. Moulton, H. T. Hutchins, H. T. Miller, Jr., T. F. Riggs, H. D. Long, L. C. Bixler, J. T. Geraghty and C. E. Brush, Jr. Norman McLeod Harris goes to Chicago to teach bacteriology, Glanville C. Rusk accepts a position with the pathologic institute of New York, and Burton D. Myers becomes teacher of anatomy at the Indiana State University.

---

THE *British Medical Journal* thus characterizes a young woman of this city: "Miss Mary C. Lowell, M.D., of Boston, is said to be the only woman in the world who is entitled to practice the professions of medicine and law by virtue of the possession of degrees in those faculties. She was the first woman assistant superintendent of the Maine State Hospital for the Insane. After holding this position for five years she visited the hospitals of various European capitals. The love of study prompted her to elect a course in law, and it is said to be her intention to obtain two more degrees—Bachelor of Jurisprudence and Master in Chemistry."—*Boston Medical and Surgical Journal.*

---

DURING the month of January last there were two cases of plague in Manila, 17 in February, 42 in March, and a rapid increase in the early part of April lead the health authorities to make a vigorous campaign against its further spread. The entire Chinese population was inoculated with Shiga's antiseptic prophylactic serum, and no Chinese was allowed to land until first submitting to such inoculation. Rat catchers caught and turned over to the board 10,821 rats, of which number about one per cent. were found to be affected with the plague. These and regular sanitary precautions kept the number of cases for the month down to 52, and the number has grown less in a satisfactory degree since.

---

AT the recent meeting of the National Dental Association at Asheville, the following resolution was adopted:

"*Resolved*, That it is the sense of the 'National Dental Association' that each medical college in the United States should include in its curriculum a lectureship on 'Oral Hygiene, Prophylaxis, and Dental Pathology.'"

The dental profession feels that with the introduction of the teaching of oral hygiene into the public schools, which they are striving to accomplish, and the cooperation of medical men who have been specially instructed on this subject, that a great stride will have been made toward the prevention of caries of the teeth, not to mention many other good results to the general system, which would surely follow a better care of the oral cavity.

---

THE following statement is made in *American Medicine:* "The autopsy on the late Dr. Laborde, of Paris, disclosed an interesting fact regarding the brain. Dr. Laborde was an exceedingly fluent speaker, and it was desired to ascertain if the speech center was unusually developed. The left inferior frontal convolution was found to be much larger than the right. Its volume was the more remarkable because the brain as a whole was small, weighing 1,234 gm. (41 oz.), and the convolutions in general were but little complicated." Interesting as such observations are, we cannot but regard with scepticism the statement that fluency in speech should have so direct a bearing upon the cerebral speech area. Valuable as such apparently positive evidence may be, it needs very decidedly in matters of this sort to be controlled by a study of many brains, both of fluent and of poor speakers.

---

THE general public, the *Scientific American* fears, is not acquainted with the dangers arising from arsenic coloring matter in wall paper. A recent death in Palmer, Mass., is directly attributed, by the medical authorities, to this cause. The trouble which resulted so disastrously made its appearance a year and a half ago in what seemed to be nervous dyspepsia. Two months of travel abroad seemed to greatly improve the patient, but on returning home he soon grew worse again. On account of certain conflicting symptoms which could not readily be accounted for, a specialist was called in and gave his opinion that there was arsenic poisoning in the system. An investigation was then made which resulted in the discovery of arsenic colors in the wall paper of the sitting room. This room had been papered shortly previous to the appearance of the first symptoms. The wall paper was at once removed, but the disease had by this time progressed so far that it was impossible to save the life of the unfortunate victim.

---

THE osteopaths are beginning to sit up and notice one another in the columns of their official organs and what has been looked for is come to pass. They are beginning to find fault with one another as to the ethics of their "profession," and are drawing fine lines of distinction as between "regular" osteopaths and "lesion" osteopaths, whatever those terms may mean to the practitioners of massage. The heretics believe there are other agencies than manipulation which will cure disease conditions, and so the squabble has begun. In discussing the matter a "Dr. C. W. Young, D.O.," says, referring to "Dr." Still's text-books on osteopathy, and be it remembered that "Dr." Still is the inventor of this method of massage:

Chapter XIV. is devoted to the treatment of diphtheria and other diseases by pouring glycerine and water in the ear. He described a desperate case of croup, where water, glycerine and a wet rag were used and "osteopathic treatment." I have heard the book ridiculed for this chapter. I do not believe in ridiculing anything until you know what you are talking about. I tried this ear treatment in the case of the surgically maimed child who died while under my treatment of diphtheria. It seemed to give relief and she begged to have it repeated. She seemed to be improving at the time she choked to death when the nurse attempted to force her to swallow too much water all at once. I am satisfied that this ear treatment is all right as well as all other treatments described by Dr. Still. Too many osteopaths are neglecting to read the Old Doctor's books. He says God and experience are his authorities. I believe in these authorities. They are far ahead of the authority of theory.

Which is chiefly interesting because it is so foolish.—*Ex.*

---

THE "poison squad," commanded by Dr. Harvey W. Wiley, chief chemist of the agricultural department, will have an opportunity to "look upon the wine when it is red." On October 1, the experiments in adulterations will be resumed. Wine is expected to figure prominently on the menu, as salicylic acid will be the preservative forming the basis of experimentation. Word has been passed around that a dozen new volunteers are desired. It is expected the prospect of wine will bring forward a rush of those willing to be sacrificed in the interest of science. During his former period of running a government boarding house, Dr. Wiley disposed of borax and formaldehyde. He will now conduct thorough and exhaustive tests with salicylic acid. Dr. Wiley said one of the leading wine experts of Europe, whom he consulted while in London, remarked "that there are no chateau or vineyard wines shipped to the United States. The American people drink but labels." Dr. Wiley thought this declaration too sweeping. He believes there are some genuine chateau and vineyard wines imported into the United States, but he believes they are usually special importations, often made for individuals, and that the general buyer seldom gets such wines. The label looks all right, and the retail dealer may honestly believe that the wine is genuine, but in most cases, it is not. Dr. Wiley is of the opinion that most of the Scotch whiskies imported into the United States are blended.

---

THE American Medical Association offers annually a gold medal, of the value of one hundred dollars, for the best essay on any subject relating to medicine or surgery. To the recipient of this prize will be given the option of the gold medal, or a bronze replica of the medal, and the balance of the appropriation (about $90.00) in money; or the entire amount ($100.00) in money. Inasmuch as the association annually sets apart the sum of five hundred dollars for original research, this prize is offered to stimulate the production of a superior practical paper based upon either experimental studies, or clinical investigation, or both. The committee will give preference to papers having the greatest brevity consistent with thorough consideration of the subject, and recommends that they do not exceed five thousand words. The committee, while not restricting the choice of subjects, recommends as an important subject for consideration the Therapeutic Value of the Digestive Ferments. Competing essays must be typewritten, and bear no mark revealing their authorship; but instead of the name of the author there must appear on each essay a motto, and accompanying each essay a sealed envelope containing the name of the author and bearing on its outer surface the motto of identification. No envelope will be opened by the committee until a decision has been reached as to the most deserving essay. The other essays will be returned to their respective authors. The committee reserves the right to reject all essays if none are found worthy of the association medal. Competing essays must be in the hands of the chairman of the committee not later than April 1, 1904. Lewis S. McMurtry, of Louisville, Ky., Chairman; Burnside Foster, of St. Paul, Minn.; M. H. Fussell, of Philadelphia, Penn., Committee.

